# Hypothermia Protects against Ischemic Stroke through Peroxisome-Proliferator-Activated-Receptor Gamma

**DOI:** 10.1155/2022/6029445

**Published:** 2022-07-14

**Authors:** Shuai Shao, Tian-Yi Lu, Jian-Song Zhang, Wen-Jun Wang, Xiao-Hua Zhang, Kui Chen, Feng Jia

**Affiliations:** ^1^Department of Neurosurgery, Ren Ji Hospital, Shanghai Jiao Tong University School of Medicine, Shanghai 200127, China; ^2^Department of Urology Surgery, Gansu Provincial Hospital, The First Clinical Medical College of Gansu University of Chinese Medicine, Lanzhou 730013, China; ^3^Department of Neurosurgery, Chang Zheng Hospital, The Second Military Medical University, Shanghai 200003, China; ^4^College of Chemistry and Chemical Engineering, Shanghai University of Engineering Science, Shanghai 201620, China; ^5^Department of Neurosurgery, Shanghai East Hospital, Tongji University School of Medicine, Shanghai 200120, China; ^6^Department of Neurosurgery, Nantong First People's Hospital, Affiliated Hospital 2 of Nantong University, Nantong 226001, China

## Abstract

Ischemic stroke (IS) remains a global public health burden and requires novel strategies. Hypothermia plays a beneficial role in central nervous system diseases. However, the role of hypothermia in IS has not yet been elucidated. In this study, we determined the role of hypothermia in IS and explored its underlying mechanisms. The IS phenotype was detected based on infarct size, infarct volume, and brain edema in mice. Neuroinflammation was evaluated by the activation of microglial cells and the expression of inflammatory genes after ischemia/reperfusion (I/R) and oxygen-glucose deprivation/reperfusion (OGD/R). Neuronal cell apoptosis, cleaved caspase-3 and Bax/Bcl-2 expressions, cell viability, and lactate dehydrogenase (LDH) release were detected after I/R and OGD/R. Blood–brain barrier (BBB) permeability was calculated based on Evans blue extravasation, tight junction protein expression, cell viability, and LDH release after I/R and OGD/R. The expression of peroxisome proliferator-activated receptor gamma (PPAR*γ*) was assessed after OGD/R. Our results suggested that hypothermia significantly reduced infarct size, brain edema, and neuroinflammation after I/R. Hypothermia increased PPAR*γ* expression in microglial cells after OGD/R. Mechanistic studies revealed that hypothermia was a protectant against IS, including attenuated apoptosis of neuronal cells and BBB disruption after I/R and OGD/R, by upregulating PPAR*γ* expression. The hypothermic effect was reversed by GW9662, a PPAR*γ* inhibitor. Our data showed that hypothermia may reduce microglial cell-mediated neuroinflammation by upregulating PPAR*γ* expression in microglial cells. Targeting hypothermia may be a feasible approach for IS treatment.

## 1. Introduction

Stroke remains the leading cause of mortality and morbidity worldwide [1], and 70% of the strokes are caused by ischemic stroke (IS) [2]. Despite advancements in clinical treatments, the Food and Drug Administration-approved treatments to IS only include endovascular thrombectomy and intravenous thrombolysis with tissue plasminogen activator (tPA) [3, 4]. Owing to the limitation of the time window and hemorrhagic risk of tPA, only a small number of patients with stroke receive effective treatment [5]. Therefore, strategies that benefit more patients with stroke are urgently required.

Neuroinflammation is the majority pathophysiological process that contributes to the development of brain injury following IS. Previous studies have shown that anti-inflammatory strategies are beneficial for IS [6]. Microglial cells are the innate immune cells in the central nervous system (CNS) that are widely activated following IS [7]. Studies have reported that activated microglial cells release proinflammatory cytokines and aggravate neuroinflammation [8, 9]. Drastic neuroinflammation enhances apoptosis of the neuronal cell and exacerbates the disruption of the tight junction between the endothelial cells [10]. Thus, targeting microglia-mediated neuroinflammation is a feasible strategy for IS treatment.

Previous studies have demonstrated the involvement of hypothermia in a variety of diseases, including stroke, traumatic brain injury, and hypoxic-ischemic encephalopathy [11–13]. Growing evidence from animal experiments and clinical research has shown that therapeutic hypothermia prevents brain injury by suppressing oxidative stress, inflammatory responses, and cell death [14]. Therefore, hypothermia is an attractive treatment option for IS. However, its underlying protective mechanism remains unclear. Peroxisome proliferator-activated receptor gamma (PPAR*γ*) modulates macrophage/microglial polarization and microglia-mediated neuroinflammation after IS [15]. However, whether hypothermia plays an important role in regulating PPAR*γ* in microglial cells after middle cerebral artery occlusion (MCAO) or oxygen-glucose deprivation/reperfusion (OGD/R) has not yet been reported.

In this study, we investigated the effect of hypothermia in IS, including infarct size, infarct volume, brain edema, neuroinflammation in vivo and vitro, neuronal cell apoptosis in vivo and vitro, and blood–brain barrier (BBB) disruption in vivo and vitro using MCAO model and OGD/R models. We also explored the cellular mechanisms and studied how hypothermia protects against ischemia and hypoxia through PPAR*γ* in microglial cells after MCAO and OGD/R.

## 2. Methods

### 2.1. Animals and Experimental Design

Adult male C57BL/6 mice (8–10 weeks, 22–25 g) were used in this study. The mice were housed in 12-hour light and 12-hour dark cycle in the standard cages. Food and water were freely available. All animal experiments were approved by the Animal Care and Experimental Committee of the School of Medicine of Shanghai Jiao Tong University.

The mice were randomly divided into four groups: sham injury with normothermia group (SNG; 37°C; *n* = 36), sham injury with hypothermia (SHG; 33°C; *n* = 36), MCAO with normothermia group (MNG; 37°C; *n* = 36), and MCAO with hypothermia group (MHG; 33°C; *n* = 36). The investigators were blinded to group assignments during all result assessments.

### 2.2. Drug Administration and Temperature Measurement

Mice brain temperature was monitored using a digital electronic thermometer (Omega Engineering, USA). Drug administration was performed as previously described [16]. Briefly, HPI-201 was dissolved in saline and injected intraperitoneally (i.p.). For MNG and MHG mice, the first injection (2 mg/kg) was administered 30 min after MCAO. The brain temperature cooled from 37°C to 33°C in approximately 30 min. The additional injections (1 mg/kg) were given 3 and 5 hours after MCAO to maintain constant mild hypothermia at 33°C for 6 hours. The brain temperature gradually returned to normothermia. For SNG and SHG mice, equivalent saline was given at the same time points, and the brain temperature was maintained at the normothermia.

### 2.3. Brain I/R Model

The I/R model was performed as previously described [17]. Briefly, the mice were anesthesia with 2% avertin (Sigma-Aldrich, USA). Depilatory cream was used to remove hair from the neck skin. Cutting neck skin alongside the midline and separated the left common carotid artery, the internal carotid artery, and the external carotid artery. The left middle cerebral artery was blocked with a 2.2 mm-coated nylon suture (0622, Yushun, China). After 60 min of occlusion, the nylon suture was removed. Mice in sham groups underwent the same procedures, but the nylon suture was not inserted into the middle cerebral artery. Mice were housed separately with free availability of food and water after the operations.

### 2.4. 2,3,5-Triphenyl Tetrazolium Hydrochloride (TTC) Staining

Experimental mice were euthanized using overdose isoflurane 24 hours after I/R. Brains were quickly removed and frozen at -20°C for 30 mins. The brains were coronally cut into 2 mm-thick sections and incubated with 2% TTC (Sigma-Aldrich, USA) at 37°C for 15 minutes. Infarct size and volume were calculated by ImageJ software (National Institutes of Health, USA).

### 2.5. Brain Water Content

Experimental mice were euthanized using overdose isoflurane 24 hours after I/R. Brains were quickly removed and divided into the contralateral hemisphere, ipsilateral hemisphere, and cerebellum. The ipsilateral hemisphere was weighed (wet weight) and placed in an oven at 105°C for 72 hours for dry weight. The brain water content was measured as follows: (wet weight − dry weight/wet weight) × 100%.

### 2.6. Neurological Score

The neurological score was assessed at 24 hours after I/R, using the Garcia score system as previously described [18]. The Garcia scores include vibrissae touch, touch of the trunk, climbing wall of the wire cage, movements of forelimbs, spontaneous movements of all limbs, and spontaneous activity. All results were assessed by an investigator blinded from the group allocation.

### 2.7. Immunofluorescence

Experimental mice were euthanized using overdose isoflurane 24 hours after I/R. Brains were quickly removed and fixed with 4% paraformaldehyde for 24 hours. Then, the samples were dehydrated and paraffin-embedded. After antigen retrieval treatment, coronal sections were incubated with a blocking buffer at 37°C for 1 hour, and the coronal sections were incubated with primary antibodies (anti-Iba1, 1 : 200, Abcam, USA; anti-NeuN, 1 : 200, Abcam, USA) at 4°C overnight. Secondary antibodies of fluorochrome-conjugated were incubated (Thermo Fisher Scientific, USA) at room temperature for 2 hours 24 hours later. Finally, the coronal sections were counterstained with DAPI (Thermo Fisher Scientific, USA). Images were captured with a fluorescence microscope (Leica, German).

### 2.8. Cell Culture and OGD/R Model

BV2 microglial cells were cultured in Dulbecco's Modified Eagle Medium (DMEM)/F12 (Thermo Fisher Scientific, USA) supplemented with 10% fetal bovine serum (FBS, Thermo Fisher Scientific, USA) and 1% penicillin-streptomycin (PS, Thermo Fisher Scientific, USA). HT-22 neuronal cells were cultured in DMEM supplemented with 10% FBS and 1% PS. bEnd.3 endothelial cells were cultured in DMEM with 10% FBS and 1% PS. For OGD treatment, the media were replaced with glucose-free EBSS (Thermo Fisher Scientific, USA), and the cell plates were placed in a hypoxia incubator (Sigma- Aldrich, USA) at 37°C with 95% N2 and 5% CO2. The cells were exposed to the OGD stimulation at 37°C for 3, 6, and 12 hours. For the control treatment, the media were replaced with EBSS (Thermo Fisher Scientific, USA) supplemented with glucose in a humidified incubator at 37°C with 5% CO2 and 95% air for the same time. After OGD treatment and control treatment, the media was replaced with normal media, and the cell plates were placed in a humidified incubator at 37°C with 5% CO2 and 95% air for 24 h reperfusion.

### 2.9. Proinflammatory Microglia Cell Model

Conditioned medium (CM) collected from HT-22 neuronal cells for 6 h OGD and bEnd.3 endothelial cells for 12 h OGD was used to stimulate BV2 microglial cells. Control BV2 microglial cells were treated with medium collected from HT-22 neuronal cells and bEnd.3 endothelial cells without OGD. During the stimulation, hypothermia (33°C) and GW9662 (20 *μ*M) were treated.

### 2.10. Microglial Cells-Neuronal Cells and Microglial Cells-Endothelial Cells Coculture

The cells were cocultured using Transwell cell culture inserts (Corning, USA). BV2 microglial cells grown on culture inserts were treated with vehicle placed in normothermia (37°C), hypothermia (33°C), CM placed in normothermia (37°C), CM placed in hypothermia (33°C), and CM supplemented with GW9662 (20 *μ*M) placed in hypothermia (33°C) for 12 hours. HT-22 neuronal and bEnd.3 endothelial cells were exposed to OGD for 6 and 12 h, respectively. The medium was then replaced with a normal medium. Cocultured cells systems were generated by adding the BV2 microglial cells inserts on top of HT-22 neuronal cells and bEnd.3 endothelial cells. The coculture systems were maintained for 24 hours before the BV2 microglial cell inserts were removed. The survival was analyzed using an MTT kit (Roche, Switzerland), and the cell death was evaluated by lactate dehydrogenase (LDH) release according to the manufacturer's instructions (Beyotime, China).

### 2.11. Quantitative RT-qPCR

Experimental mice were euthanized using overdose isoflurane 24 hours after I/R. TRIzol (Thermo Fisher Scientific, USA) was used to extract total RNA from the ipsilateral hemisphere, and cDNA was synthesized using reverse transcription kits (Takara, Japan) following the manufacturer's instructions. RT-qPCR was conducted using SYBR Green Mix (Thermo Fisher Scientific, USA) on a LightCycler 480 II (Roche, Switzerland). Gene expression was normalized by GAPDH. Primer sequences were as follows (Table 1).

### 2.12. Western Blotting Analysis

Experimental mice were euthanized using overdose isoflurane 24 hours after I/R. The ipsilateral hemisphere was collected and lysed in lysis buffer containing 1 mM PMSF and protease inhibitors cocktail. The proteins were separated by 8% or 10% SDS-PAGE and transferred to PVDF membranes. The membranes were incubated with blocking buffer for 1 hour, then incubated with primary antibodies at 4°C overnight, followed by incubation with secondary antibodies for 2 hours at room temperature 12 hours later. The membranes were detected with chemiluminescence substrates (Thermo Fisher Scientific, USA). The gray values of immunoreactive bands were calculated with ImageJ software. Proteins level were normalized by *α*-tubulin or GAPDH. The following primary antibodies were used: anti-Iba1 (1 : 1000, Abcam, USA), anti-PPAR*γ* (1 : 1000, Abcam, USA), anti-Cleaved Caspase-3 (1 : 1000, Abcam, USA), anti-Bcl-2 (1 : 1000, Abcam, USA), anti-Bax (1 : 1000, Abcam, USA), anti-Occludin (1 : 500, Santa Cruz, USA), and anti-GAPDH (1 : 1000, Cell Signaling Technology, USA).

### 2.13. Measurement of EB Extravasation

EB (2%, Sigma-Aldrich, USA) was intravenously injected (5 ml/kg) 23 hours after I/R. The dye was circulated for 1 hour. Collected and weighed the ipsilateral hemisphere, then homogenized samples in 1 ml ice-cold PBS. The supernatants were harvested, and an equal volume of 100% trichloroacetic acid (Thermo Fisher Scientific, USA) was added. The mixture was incubated at 4°C overnight. The supernatants were measured at a wavelength of 610 nm by spectrophotometry. The concentration of EB extravasation in the ipsilateral hemisphere was calculated using a standard curve.

### 2.14. Statistical Analysis

Data were expressed as mean ± SD. Statistical analysis was performed using Prism 8.0 (GraphPad Software, USA). Statistical differences were assessed by Student's *t*-test for two-group comparisons or ANOVA followed by Tukey's test for multiple comparisons among more than two groups. *P* ≤ 0.05 was considered statistically significant.

## 3. Results

### 3.1. Hypothermia Protects against Ischemia/Reperfusion (I/R) in Mice

**Figure 1:**
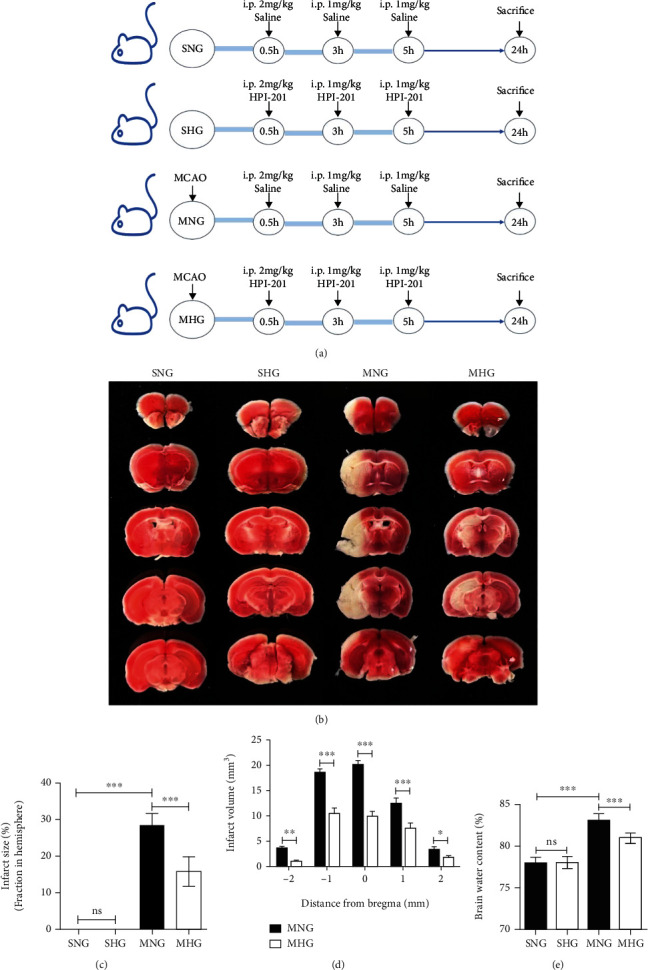
Hypothermia protects against I/R in mice. (a) The animals and experimental designs. (b) Representative TTC staining in cerebral sections for every group. (c) Quantification of infarct size in total brain hemisphere. (d) Quantification of infarct volume in serial coronal sections. (e) Quantification of brain water content in ipsilateral brain tissues for every group. Values are presented as Mean ± SD, *n* = 6 per group. ns: not significant. ^∗^*P* < 0.05, ^∗∗^*P* < 0.01, ^∗∗∗^*P* < 0.001.

To examine the effect of hypothermia in IS, we generated SHG and MHG mice by using the neurotensin receptor 1 agonist HPI-201. The animals and experimental designs are shown in Figure 1(a). Mice in different groups were subjected to a 60-minute I/R or sham operation. The infarct size, infarct volume, and brain water content for each group were analyzed for the next 24 hours. Measurements of infarct size and infarct volume by 2,3,5-triphenyltetrazolium chloride stating showed that MCAO induced infarction size and infarction volume in both groups, but was significantly less in the MHG mice than in the MNG mice (Figures 1(b)–1(d)). Consistently, brain water content measurements showed that brain edema caused by MCAO was significantly lower in the MHG mice than in the MNG mice (Figure 1(e)).

### 3.2. Hypothermia Inhibits the Activation of Microglia/Macrophage and Neuroinflammation after I/R in Mice

**Figure 2:**
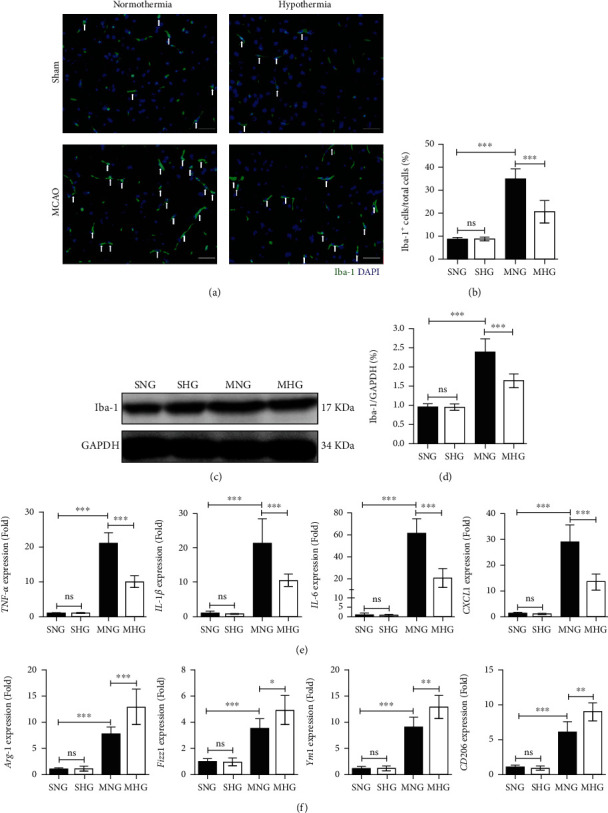
Hypothermia inhibits activation of microglia/macrophage and neuroinflammation after I/R in mice. (a) Representative immunofluorescence staining of Iba-1+ in the ischemic region for every group. Scale bar: 50 *μ*m, magnification: 400×. (b) Quantification analyses of the percentage of Iba-1 positive cells in total cells. (c) Representative western blotting analysis of Iba-1 in the ischemic region for every group. GAPDH was used as a loading control. (d) Quantifications of the expression of Iba-1. (e) qRT-PCR analysis of M1 activated microglia/macrophage markers in the ipsilateral brain tissues for every group. (f) qRT-PCR analysis of M2 activated microglia/macrophage markers in the ipsilateral brain tissues for every group. All gene expressions were normalized to GAPDH. Values are presented as Mean ± SD, *n* = 6 per group. ns: not significant. ^∗^*P* < 0.05, ^∗∗^*P* < 0.01, ^∗∗∗^*P* < 0.001.

The activation of microglia/macrophage and microglia/macrophage-mediated neuroinflammation in ischemic brain tissues for every group was analyzed 24 hours after I/R or sham operation. Immunofluorescence staining showed that I/R induced more Iba-1+ cells in the ischemic region in the MNG and MHG mice than in the SNG mice and SHG mice (Figures 2(a) and 2(b)). Significantly fewer Iba-1+ cells were detected in the MHG mice than in the MNG mice (Figures 2(a) and 2(b)). Western blotting analysis showed a significantly lower protein level of Iba-1 in the ischemic region of the MHG mice than in the MNG mice (Figures 2(c) and 2(d)). The results of quantitative real-time polymerase chain reaction (qRT-PCR) analysis showed that drastic neuroinflammation was detected in the MNG and MHG mice (Figures 2(e) and 2(f)). The expression of proinflammatory cytokines, including TNF-*α*, IL-1*β*, IL-6, and CXCL1, were substantially lower in the MHG mice than in the MNG mice (Figure 2(e)). Consistently, the expression of anti-inflammatory cytokines, including Arg-1, Fizz-1, Ym-1, and CD206, was higher in the MHG mice than in the MNG mice (Figure 2(f)).

### 3.3. Hypothermia Enhances the Protein Level of PPAR*γ* in BV2 Microglial Cells and Promotes Microglial Cells Polarized from Proinflammatory Phenotype to Anti-Inflammatory Phenotype through PPAR*γ* after OGD/R

**Figure 3:**
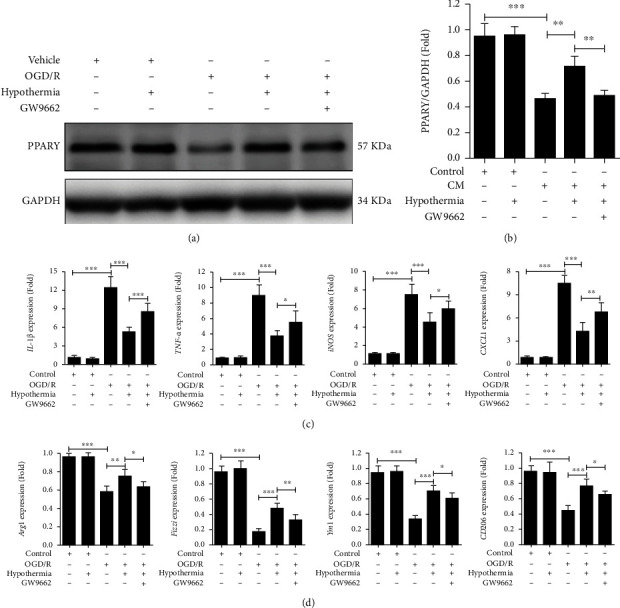
Hypothermia enhances the protein level of PPAR*γ* in BV2 microglial cells and promotes microglial cells polarized from proinflammatory phenotype to anti-inflammatory phenotype through PPAR*γ* after OGD/R. (a) Representative western blotting analysis of PPAR*γ* in BV-2 microglial cells 24 hours after with or without 3 h-OGD for every group. Hypothermia and GW9662 (20 *μ*M) were treated in the next 24 hours of reperfusion, respectively. GAPDH was used as a loading control. (b) Quantifications of the expression of PPAR*γ*. (c) qRT-PCR analysis of M1-activated microglial cell markers 24 hours after with or without 3 h-OGD for every group. Hypothermia and GW9662 (20 *μ*M) were treated in the next 24 hours of reperfusion, respectively. (d) qRT-PCR analysis of M2 activated microglial cells markers 24 hours after with or without 3 h-OGD for every group. Hypothermia and GW9662 (20 *μ*M) were treated in the next 24 hours of reperfusion, respectively. All gene expressions were normalized to GAPDH. Experiments were repeated for 4 times. Values are presented as Mean ± SD. ns: not significant. ^∗^*P* < 0.05, ^∗∗^*P* < 0.01, ^∗∗∗^*P* < 0.001.

The protein level of PPAR*γ* in BV-2 microglial cells for every group were analyzed 3 h OGD/R or control treatment 24 h later. Western blotting analysis showed a significantly lower protein level of PPAR*γ* in OGD/R (Figures 3(a) and 3(b)). Hypothermia increased PPAR*γ* protein expression in BV2 microglial cells after OGD/R (Figures 3(a) and 3(b)). However, GW9662 (20 *μ*M), an inhibitor of PPAR*γ*, reversed the effect of hypothermia (Figures 3(a) and 3(b)). The results of the qRT-PCR analysis showed that the proinflammatory cytokines, including IL-1*β*, TNF-*α*, iNOS, and CXCL1, were substantially increased after OGD/R, and hypothermia reduced the mRNA expressions of proinflammatory cytokines after OGD/R. However, GW9662 reversed the function of hypothermia (Figure 3(c)). The results of qRT-PCR analysis showed that the levels of anti-inflammatory cytokines, including Arg-1, Fizz-1, Ym-1, and CD206, were substantially decreased after OGD/R, and hypothermia enhanced the mRNA expressions of anti-inflammatory cytokines after OGD/R (Figure 3(d)). Consistently, the effect of hypothermia was converted by GW9662 (Figure 3(d)).

### 3.4. Hypothermia Reduces Apoptosis of Neuronal Cells and Suppresses the Neurotoxic Effect of Proinflammatory BV2 Microglial Cells on Cocultured HT-22 Neuronal Cells through PPAR*γ*

**Figure 4:**
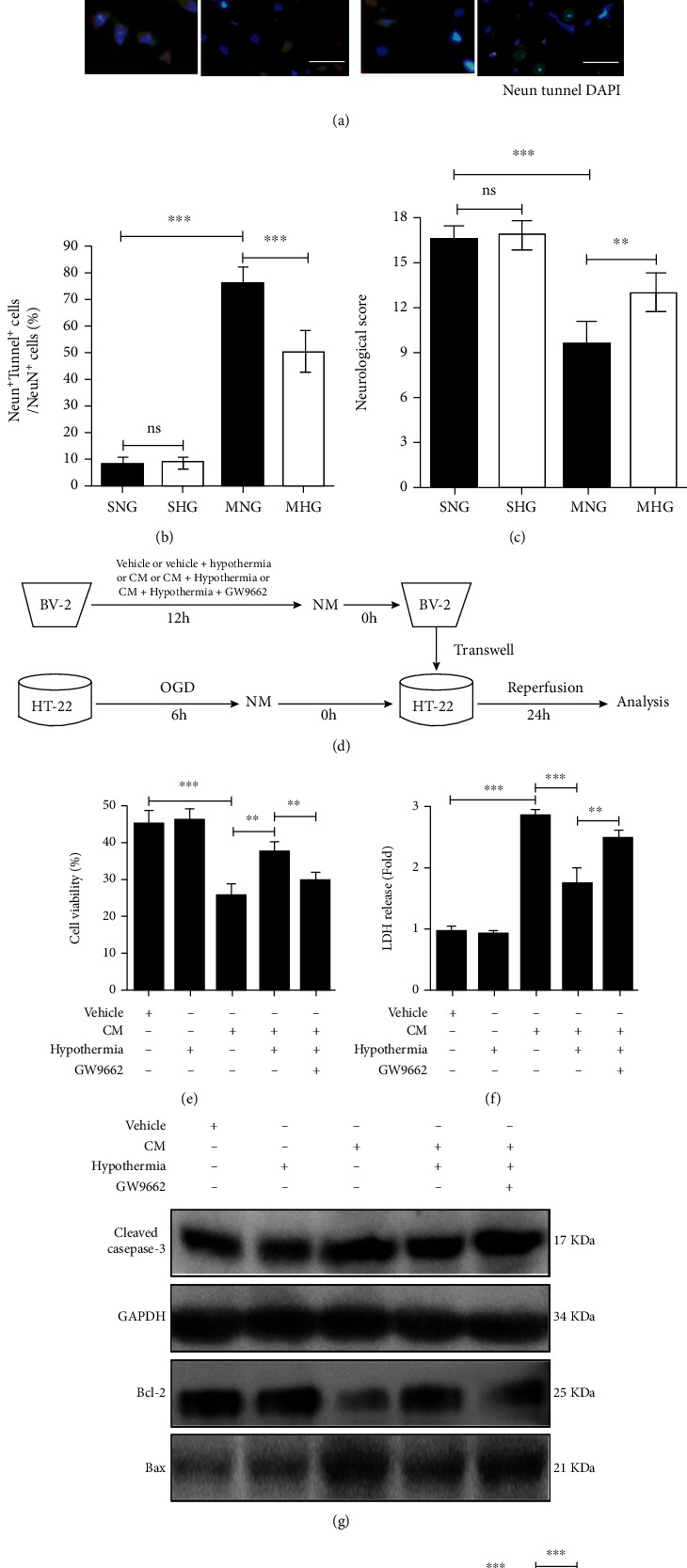
Hypothermia reduces apoptosis of neuronal cells and suppresses the neurotoxic effect of proinflammatory BV2 microglial cells on cocultured HT-22 neuronal cells through PPAR*γ*. (a) Representative Tunnel staining of neuronal cells in the ischemic region for every group. Magnifications of the boxed areas are shown by the inset images. Scale bar: 50 *μ*m. Magnification: 400×. (b) Quantitative analyses of the percentage of NeuN+ Tunnel+ cells in NeuN+ cells. (c) Neurological scores for every group. (d) The strategy of the cocultured system. (e) Quantifications of cell ability for every group. (f) Quantifications of LDH release ratio for every group. (g) Representative western blotting analysis of Cleaved Caspase-3, Bcl-2, and Bax for every group. GAPDH was used as the loading control. (h) Quantifications of the expression of Cleaved Caspase-3. (i) Quantifications of the expression ratio of Bcl-2/Bax. *n* = 6 per group for (a)–(c). Experiments were repeated for 4 times for (e)–(i). Values are presented as Mean ± SD. ^∗^*P* < 0.05, ^∗∗^*P* < 0.01, ^∗∗∗^*P* < 0.001.

The apoptosis of neuronal cells in the ischemic region for every group was analyzed 24 h after I/R or sham operation. Tunnel staining showed that I/R led to more NeuN+ Tunnel+ cells in the ischemic region in the MNG and MHG mice than in the SNG and SHG mice (Figures 4(a) and 4(b)). Significantly fewer NeuN+ Tunnel+ cells were detected in the MHG mice than in the MNG mice (Figures 4(a) and 4(b)). Similarly, the MHG mice manifested significantly higher neurological scores than the MNG mice (Figure 4(c)). To further confirm that hypothermia indirectly reduced neuronal cells death by regulating PPAR*γ* activation in microglial cells, a microglia-neuron coculture system was established (Figure 4(d)). The results showed that the CM of proinflammatory BV2 microglial cells enhanced post-OGD/R HT-22 neuronal cells death, as indicated by decreased cell viability and increased LDH release, which was alleviated in hypothermia-treated CM of proinflammatory BV-2 microglial cells, and GW9662 reversed the effect of hypothermia (Figures 4(e) and 4(f)). Western blotting analysis demonstrated that the CM of proinflammatory BV2 microglial cells significantly enhanced the protein level of Cleaved Caspase-3 and reduced the protein ratio of Bcl-2/Bax in post-OGD/R HT-22 neuronal cells (Figures 4(g)–4(i)). The protein expression of Cleaved Caspase-3 was lower, and the protein ratio of Bcl-2/Bax was higher in post-OGD/R HT-22 neuronal cells with CM of hypothermia-treated proinflammatory BV-2 microglial cells, in which GW9662 blocked the function of hypothermia (Figures 4(g)–4(i)).

### 3.5. Hypothermia Attenuates BBB Disruption and Alleviates the Disruption of Proinflammatory BV-2 Microglial Cells on Cocultured bEnd.3 Endothelial Cells through PPAR*γ*

**Figure 5:**
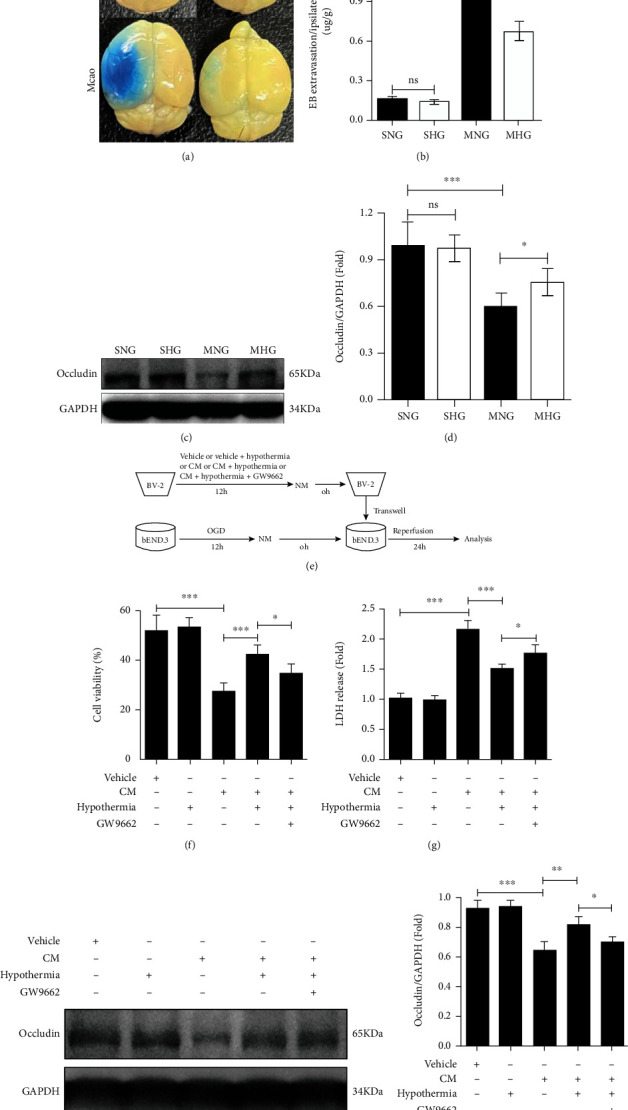
Hypothermia attenuates BBB disruption and alleviates the disruption of proinflammatory BV-2 microglial cells on cocultured bEnd.3 endothelial cells through PPAR*γ*. (a) Representative photographs of EB extravasation for every group. (b) Quantifications of EB extravasation. (c) Representative western blotting analysis of Occludin in the ischemic region for every group. GAPDH was used as a loading control. (d) Quantifications of the expression of Occludin. (e) The strategy of the cocultured system. (f) Quantifications of cell ability for every group. (g) Quantifications of LDH release ratio for every group. (h) Representative western blotting analysis of Occludin for every group. GAPDH was used as a loading control. (i) Quantifications of the expression of Occludin. *n* = 6 per group for (a)–(d). Experiments were repeated for 4 times for (f)–(i). Values are presented as Mean ± SD. ^∗^*P* < 0.05, ^∗∗^*P* < 0.01, ^∗∗∗^*P* < 0.001.

BBB disruption in ischemic brain tissues for every group was analyzed 24 h after I/R or sham operation. Evans blue (EB) staining showed that I/R induced EB extravasation in ipsilateral hemispheres of the MNG and MHG mice, but significantly less in the MHG mice than in the MNG mice (Figures 5(a) and 5(b)). Consistently, western blotting analysis showed a significantly lower protein level of Occludin in the ischemic region of the MHG and MNG mice than in the SNG and SHG mice, and the expression of Occludin was higher in the MHG mice than in the MNG mice (Figures 5(c) and 5(d)). To determine whether hypothermia indirectly alleviates endothelial cells disruption by modulating PPAR*γ* activation in microglial cells, a microglia-endotheliocyte coculture system was used (Figure 5(e)). The results showed that proinflammatory BV2 microglial cells increased post-OGD/R bEnd.3 endothelial cell death, as suggested by decreased cell viability and increased LDH release, which was attenuated by hypothermia, and GW9662 reversed the effect of hypothermia (Figures 5(f) and 5(g)). Western blotting analysis illustrated that the CM of proinflammatory BV2 microglial cells significantly decreased the protein level of Occludin in post-OGD/R bEnd.3 endothelial cells (Figures 5(h) and 5(i)). The protein expression of Occludin was higher in post-OGD/R bEnd.3 endothelial cells with CM of hypothermia-treated proinflammatory BV-2 microglial cells, in which GW9662 inhibited the impact of hypothermia (Figures 5(h) and 5(i)).

## 4. Discussion

Although hypothermia plays beneficial roles in various diseases, its cellular mechanism can be multifaceted, and its underlying cellular mechanism remains unclear. In this study, we demonstrated for the first time that hypothermia was a significant protectant against I/R and OGD/R by upregulating PPAR*γ* activation in microglial cells.

Previous studies have suggested that hypothermia can be effective in the treatment of cerebrovascular diseases. For example, hypothermia attenuates neuroinflammation by modulating microglia/macrophage polarization by upregulating the expression of IRF4 after MCAO [19]. Hypothermia inhibits microglial cells activation by modulating autophagy/apoptosis and the MyD88-dependent TLR4 signaling pathway after traumatic brain injury [20]. Moreover, hypothermia reduces death or disability in infants with moderate-to-severe hypoxic–ischemic encephalopathy in the near term [21]. Our results provide new evidence that hypothermia treatment is a feasible strategy for treating IS via the PPAR*γ* signaling pathway.

Suppression of neuroinflammation may be the fundamental mechanism underlying the protective effects on IS [22]. Microglia/macrophages are widely infiltrated and activated by damage-associated molecular patterns released from injured neuronal cells and subsequently mediate drastic neuroinflammation during the acute stage of IS [23]. Previous studies have reported that microglial cell deficiency inhibits the microglial cell-mediated neuroinflammation, ultimately leading to the alleviation of IS in mouse models [24]. Our data showed that hypothermia suppressed the activation of microglia/macrophage and shifted the polarization of microglia/macrophage from proinflammatory phenotype to anti-inflammatory phenotype.

Activated microglial cells produce proinflammatory cytokines such as TNF-*α*, IL-1*β*, and IL-6 during the acute phase of IS [25]. Apoptosis of neuronal cells and disruption of endothelial cells following ischemia and hypoxia are the major causes of pathological processes in IS [26]. Cytokines can cross-talk with neuronal cells and endothelial cells, aggravating the apoptosis of neuronal cells and disrupting endothelial cells [27]. Previous studies have illustrated that rosiglitazone inhibits MCAO-induced microglial cell-mediated neuroinflammation and reduces apoptosis of neuronal cells [28]. Integrin a5b1 inhibition by ATN-161 reduces neuroinflammation and attenuates BBB disruption, ultimately decreasing infarct size [29]. Our data showed that PPAR*γ* is a vital regulator of the connection between microglial cell-mediated neuroinflammation and apoptosis of neuronal cells or endothelial cells in hypothermia. PPAR*γ* is upregulated during hypothermia and plays an essential role in reducing neuronal cells apoptosis and endothelial cells disruption. More in-depth studies are required to determine how PPAR*γ* inhibits neuroinflammation during hypothermia after I/R and OGD/R.

Transcriptional regulation is an essential biological process in cells. PPARs are ligand-activated transcription factors that regulate genes essential for various biological processes [30]. PPAR*γ* can inhibit transcription factors, such as Stat 1 and nuclear factor-kB [31, 32]. It regulates the activation of microglia/macrophages by increasing the release of anti-inflammatory factors and decreasing the release of proinflammatory factors [33]. Previous studies have suggested that rosiglitazone, an agonist of PPAR*γ*, alleviates neuroinflammation through PPAR*γ*-independent mechanisms, ultimately leading to IS protection in a mouse model [34]. In this study, we demonstrated that hypothermia was a protectant against IS by activating PPAR*γ* in microglial cells. Future studies investigating the function of PPAR*γ* in hypothermia may provide mechanistic insights into IS treatment.

In summary, hypothermia plays a beneficial role in IS treatment through its downregulation of neuroinflammation, apoptosis of neuronal cells, and disruption of endothelial cells through the upregulation of PPAR*γ* in microglial cells. These data have unveiled the effects of hypothermia in the setting of IS and explored the underlying mechanisms.

## 5. Conclusion

In the present study, microglia/macrophages were activated by I/R and OGD/R, and hypothermia attenuates the number of activated microglia/macrophage and microglia/macrophage-mediated neuroinflammation by upregulating PPAR*γ* expression. In addition, hypothermia reduces neuronal cell apoptosis and attenuates BBB disruption via PPAR*γ*. This study preliminarily identifies a possible mechanism for the participation of PPAR*γ* in the neuroprotective effect of hypothermia and provides a new method for the treatment of IS. Further studies are required to explore the effect of hypothermia on PPAR*γ* after IS and the underlying mechanisms.

## Figures and Tables

**Table 1:** Primer sequence for RT-qPCR.

Gene	Sequences	Species
TNF-*α* F	5′-CCCTCACACTCAGATCATCTTCT-3′	Mouse
TNF-*α* R	5′-GCTACGACGTGGGCTACAG-3′	Mouse
IL-1*β* F	5′-GCAACTGTTCCTGAACTCAACT-3′	
IL-1*β* R	5′-ATCTTTTGGGGTCCGTCAACT-3′	Mouse
IL-6 F	5′-TAGTCCTTCCTACCCCAATTTCC-3′	
IL-6 R	5′-TTGGTCCTTAGCCACTCCTTC-3′	Mouse
CXCL1 F	5′-CTGGGATTCACCTCAAGAACATC-3′	
CXCL1 R	5′-CAGGGTCAAGGCAAGCCTC-3′	Mouse
iNOS F	5′-GTTCTCAGCCCAACAATACAAGA-3′	
iNOS R	5′-GTGGACGGGTCGATGTCAC-3′	Mouse
Arg-1 F	5′-CTCCAAGCCAAAGTCCTTAGAG-3′	
Arg-1 R	5′-AGGAGCTGTCATTAGGGACATC-3′	Mouse
Fizz-1 F	5′-CCAATCCAGCTAACTATCCCTCC-3′	
Fizz-1 R	5′-ACCCAGTAGCAGTCATCCCA-3′	Mouse
Ym-1 F	5′-CAGGTCTGGCAATTCTTCTGAA-3′	
Ym-1 R	5′-GTCTTGCTCATGTGTGTAAGTGA-3′	Mouse
CD206 F	5′-CTCTGTTCAGCTATTGGACGC-3′	
CD206 R	5′-CGGAATTTCTGGGATTCAGCTTC-3′	Mouse
GAPDH F	5′-AGGTCGGTGTGAACGGATTTG-3′	
GAPDH R	5′-TGTAGACCATGTAGTTGAGGTCA-3′	

## Data Availability

The data used to support the findings of this study are available from the corresponding author upon reasonable request.
